# Effects of Haptic Information on the Perception of Dynamic 3-D Movement

**DOI:** 10.1371/journal.pone.0106633

**Published:** 2014-09-02

**Authors:** Hiroyuki Umemura

**Affiliations:** Health Research Institute, National Institute of Advanced Industrial Science and Technology (AIST), Ikeda, Osaka, Japan; Birkbeck, University of London, United Kingdom

## Abstract

This study examined effects of hand movement on visual perception of 3-D movement. I used an apparatus in which a cursor position in a simulated 3-D space and the position of a stylus on a haptic device could coincide using a mirror. In three experiments, participants touched the center of a rectangle in the visual display with the stylus of the force-feedback device. Then the rectangle's surface stereoscopically either protruded toward a participant or indented away from the participant. Simultaneously, the stylus either pushed back participant's hand, pulled away, or remained static. Visual and haptic information were independently manipulated. Participants judged whether the rectangle visually protruded or dented. Results showed that when the hand was pulled away, subjects were biased to perceive rectangles indented; however, when the hand was pushed back, no effect of haptic information was observed (Experiment 1). This effect persisted even when the cursor position was spatially separated from the hand position (Experiment 2). But, when participants touched an object different from the visual stimulus, this effect disappeared (Experiment 3). These results suggest that the visual system tried to integrate the dynamic visual and haptic information when they coincided cognitively, and the effect of haptic information on visually perceived depth was direction-dependent.

## Introduction

Our sensory system receives information through multiple sensory modalities. Accordingly the brain must integrate information from these various sensory sources in order to perceive unified event or object. Questions surrounding this cross-modal integration process have long been a focus of study. In particular, the integration of haptic and visual information has interested many researchers. For instance, a classic study of integration of haptic and visual information, reported by Rock and Victor [1], found that visual information exerted a strong, indeed, dominant, influence over the haptic information. They investigated percepts of object size using simultaneous haptic (grasping) and visual reactions (viewing) to stimuli. In some conditions, visual size was distorted by a lens to render a conflict between these two kinds of information; Rock and Victor [1] found that the conflict between visual and tactile size resolves in favor of visual size. Such a dominance effect for visual information ('visual capture') has also been reported by others [2], [3]. However, several investigations report a situation wherein haptic information dominates visual information; this appears to happen when visual information is less reliable than the haptic information [4]–[7]. For example, Heller [4] used a stimulus whose visual information was blurred (via use of stained glass); this successfully reduced the contribution of visual information to approximate the salience level of haptic information.

Recently, integration of information from multiple modalities has been explained within the framework of statistical optimization. In this context, information from different senses are often combined in a statistically optimal fashion to obtain a more precise estimation than that derived from either sense alone [5], [8]–[10]. Specifically, this entails weighted averaging of estimations of various sensory signals; weights are then assigned according to reliability of each cues in this framework [11]–[13]. For example, Ernst and Banks [9] had participants estimate the width of stimuli defined by haptic and visual information and the reliability of visual information was manipulated through adding noise. Their results showed that the haptic information became dominant and the weight for the visual information reduced as the reliability of visual information declined. Moreover, they showed that their results were well predicted by a simulation based on Maximum-likelihood estimates (MLE), which provides optimal estimation indexed by minimized variance.

Most previous research on integration of haptic and visual information has focused on effects of interactions on participants' perception of a static object. However, several researchers have investigated the integration of multiple sensory information using dynamic events. Soto-Faraco and Kingstone [14] proposed that a majority of ordinary tasks in daily life involve dynamic information, including changing information from multiple modalities, and that these contexts should provide an advantage particularly for perception of motion and directions of motion trajectories because motion signals from single modality tend to be ambiguous in natural environments. Experimentally, their findings showed that apparent motion stream presented in one modality influences the perception of an apparent motion trajectory given to another modality. Such outcomes can be observed using any combinations of audio, visual and tactile signals. In general, these data indicate that visual input is more influential than input from other sensory modalities, however, they also suggest that the visual dominance is not inevitable. Rather the relation between different modalities can be influenced by various factors such as the distribution of attention, and/or the appropriateness of the modality for the particular task.

In the present study, I focused on the effect of dynamic haptic (proprioceptive) information on 3-D visual perception. A series of studies concerned with integration of dynamic signals, as exemplified by Soto-Faraco et al. [14], [15], reveals that integration of dynamic information emerges from various combinations of different modalities. However, this research has focused mainly on perceived directions of 2-dimensional motion that exclude the depth dimension. Yet, dynamic information also arises in 3-dimensional depth motions. There is no evidence that shows the integration of haptic and visual information for 3-D perception under a dynamic situation. There have been only a few studies on the effect of multimodal interaction on the 3-D perception. One study conducted by Kitagawa and Ichihara [16] reported that the adaptation to visual depth movement induces illusory movement of a steady sound. Here, I examined whether the dynamic haptic information could have an effect on the dynamic 3-D visual perception. For this purpose, I prepared a following situation. A participants touches (and watches) a visible surface which protrudes (becomes convex) or indents (i.e., becomes concave). Simultaneously, the force of pulling-away of push-back produced by a haptic device is given. If haptic information has an effect on the visual perception, then the visually perceived direction of depth movement would be biased toward the direction indicated by haptic information.

## Experiment 1

### Materials and Method

#### Ethics Statement

All the experimental procedures were approved by the Ethics Committee for Human and Animal Research of the National Institute of Advanced Industrial Science and Technology (AIST). Written informed consent was obtained from all the participants before the experiment.

#### Participants

Seven participants, five males and two females aged between 20–39 years, with normal or corrected-to-normal vision participated. With the exception of one participant, none knew the aim of the present experiment. All participants were right-handed.

#### Apparatus and Stimuli

**Figure 1:**
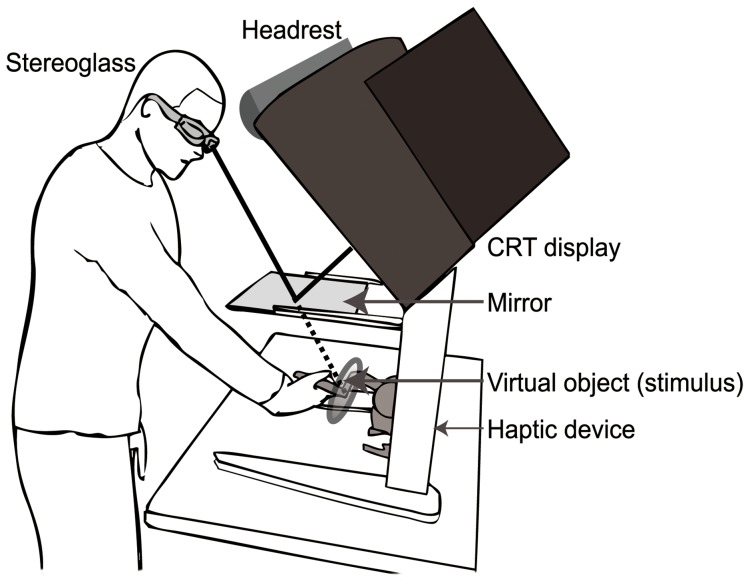
Illustration of experimental setup. Participants binocularly viewed a stimulus in a mirror that reflected the CRT display situated above the mirror which enabled the cursor position in a simulated 3-D space to coincide with the point of the stylus on a haptic device located under the mirror.

Figure 1 shows the experimental setup of the present experiment. Participants binocularly viewed a stimulus in a mirror that reflected the CRT display (Viewsonic E90fb, 19 inch, 1024×768, 120 Hz refresh rates, 60 Hz for each of eyes) situated above the mirror. The mirror was used so that the cursor position in a simulated 3-D space would coincide with the point of the stylus on a haptic device located under the mirror. The haptic stimulus was presented using a PHANToM Desktop haptic device (Sensable Technologies Inc.). The position of the PHANToM's stylus was indicated by a cursor in the shape of a blue cone. The height of the cone was 1 cm and the radius of its base circle was 0.5 cm in the simulated 3-D space. The apex of the cone coincided with the spatial position of the point of the stylus, and its axis orientation was synchronized with the orientation of the stylus. The observer's hand was beneath the mirror and could not be observed by the participant. The distance between an observer's eye and the display through the reflection on the mirror was 70 cm. CrystalEyes (StereoGraphics Inc.) liquid-crystal shutter glasses were used to present binocular disparity.

**Figure 2:**
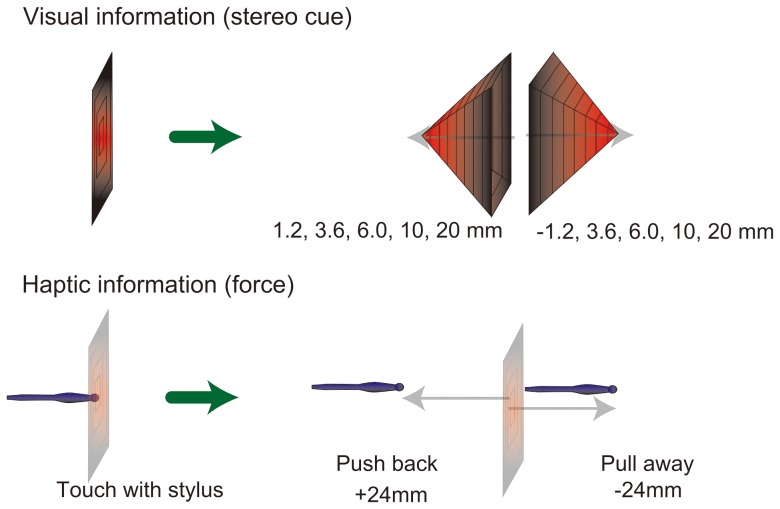
Illustration of stimuli. The visual stimuli and force stimuli were concurrently given to participants. By touching plane, force stimulus was given and visual stimulus changed synchronously. Change ended when stylus moved 24 mm and participants were required to move 24 mm in about 250 msec. Visual height and direction of force were independently determined.

Direction of hand-movement ( = direction of force) and height of a visual stimulus were independently controlled in the present experiment (Figure 2). A force stimulus was generated to move the hand of an observer when the observer touched the center of a visual stimulus (describe below). The force was a simulated spring-force. In a “pull-away condition”, the force pulled the observer's hand away from the observer perpendicularly from the contact point. While in a “push-back condition”, the force pushed observer's hand back. The anchor of the force was placed at +45 mm or −45 mm from a contact point, but the force stopped when the point of the stylus moved +−24 mm from a contact point. The force was calculated by the following equation; force (F) = 0.2 * (moved distance of stylus (mm) – 45 (mm)) N, and if F>4N, F = 4N (where N is newton). In addition to push-back and pull-away conditions, a no-move (i.e., no force) haptic condition was included. In this condition, the stylus did not move and the visual height automatically changed. The stimuli automatically changed in height 250 ms after the contact point was touched, but no force was given to the stylus. The duration of 250 ms was the time required to move the stylus 24 mm until the stimulus disappeared, in both the push-back and pull-away conditions.

The visual stimulus was a square pyramid viewed from above. At the start of each trial, the stimulus was a flat concentric square. Inside the largest square, there were seven evenly spaced inner squares. The width of the largest square was 80 mm, and was 4.4 deg at a viewing distance of 70 cm. The 3-D height of the visual stimulus dynamically changed along with the moved distance of the stylus position from the contact point perpendicular to the square. This height was defined by binocular disparity at the edge of each square. Different disparities were defined from the inner to outer square; thus, change in height resulted in a pyramid protruding toward the observer or was concaved. The smooth change in height was given by the animation of edges. The height of the innermost square attained the maximum height when the point of the stylus moved +−24 mm from a contact point. The visual height of the innermost square was determined from +−1.2, 3.6, 6, 10, 20 mm in which the positive values indicate that the surface protruded toward the observer (Figure 2).

Colors of the concentric squares were red with different lightness which became gradually darker from inner to outer, and the colors increased lightness with the height change. This change was implemented to inform subjects about the period of the height change. Without this color change, there was a case that subject did not notice a change of height in the no-move condition with small height change.

#### Procedure

Experiments were conducted in a dimly lit room. Participants could choose to stand up or sit down on a height-adjustable chair. Participants were asked to put their forehead on a headrest placed on the upper side of the CRT (see Figure 1).

At the start of each trial, participants were asked to grasp the stylus and pull it toward nearside of them. Then a concentric square appeared on the display and participants moved the cursor to touch the center of the square. Once the cursor contacted the center of the square, a force was generated except the no-move condition. When the force was given, participants were required to follow the force and to seamlessly move the stylus 24 mm in about 250 msec.

The square pyramid defined by binocular disparity protruded or indented simultaneously with the stylus movement after force generation. The visual stimulus then disappeared after the stylus moved 24 mm perpendicular to the contact position. The participants made a forced-choice response indicating whether the surface “visually protruded" or "visually concaved" by pressing a '4' key or '6' key on a ten-keyboard. Participants were instructed to ignore the direction of the force stimuli when they made a response. No feedback on their response was given.

In each session of Experiment 1, all combinations of three movement types (push-toward, pull-away, and no-move) and ten visual heights were presented twice. Each participant performed four sessions, meaning that participants served in each condition eight times. For several participants, the number of trials for no-move condition was reduced to four in order to attenuate fatigue. A rest break was provided after two sessions were completed; otherwise the entire series of sessions lasted approximately 30 minutes. The order of the conditions (combining movement type and visual heights) was randomized within each session and for each of the participants.

Before the session, participants performed practice trials. In the practice, participants first learned what kind of visual stimuli would be given with 20 no-move trials, and how the force would be given with at least 10 pull-away and push-toward trials.

### Results and Discussion

**Figure 3:**
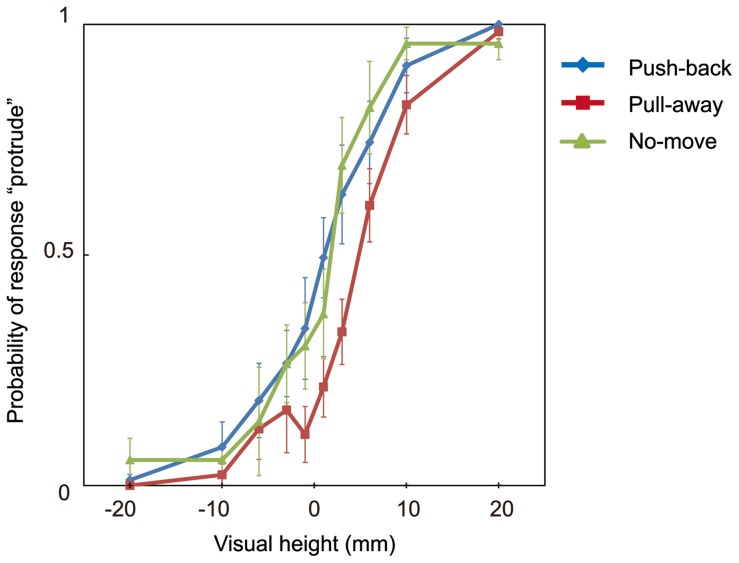
Results of Experiment 1. Vertical axis represents mean probability of participants in each condition who visually perceived stimulus protruding toward an observer as a function of height. Three conditions on the direction of hand movement are plotted. Error bars indicate mean standard errors among participants.

Averaged probabilities of stimuli judged 'protrude' over all participants are shown in Figure 3 (for all probabilities for every participant, see Table S1 in Supplementary Information). In the calculation of probabilities, responses from trials in which participants took less than 150 ms or longer than 400 ms to move the stylus 24 mm were omitted. The average number of omitted trails over participants in the push-back condition was 2.6 (3.2% of all the trials, SD = 3.5); this value was 3.0 (3.8% of all of the trials, SD = 3.3) in the pull-away condition.

**Figure 4:**
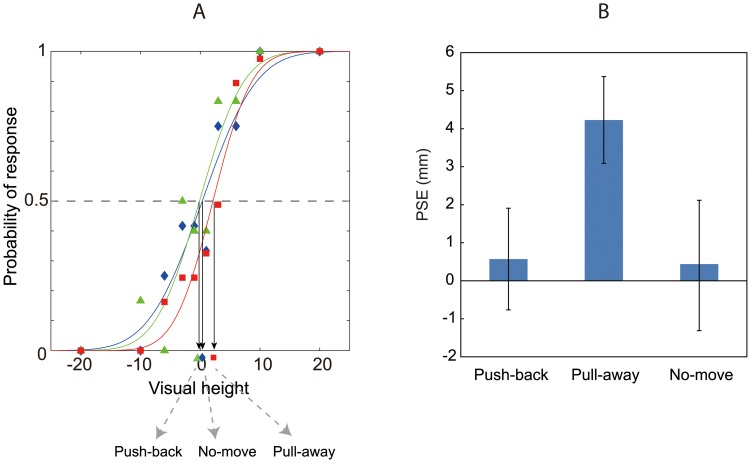
Example of curve fitting and Mean of PSEs. An example of curve fitting for the result from one participant is shown in (A). Points of subjective equality (i.e., the visual height perceived as flat) are derived from the fitted curves for each of participants. Mean of PSEs over all participants obtained from results of Experiment 1 is shown in (B). Error bars indicate mean standard errors among participants.

To quantitatively evaluate the effect of the haptic information, I fit a psychometric function to each individual's data and estimated the point of subjective equality (PSE) for each participant. To obtain psychometric functions, the data were fitted with cumulative Gaussian function by maximum likelihood estimation methods using Matlab (MathWorks). Figure 4A shows an example fit of the results from one participant. Mean PSEs over observers for each condition (of movement) in Experiment 1 appear in Figure 4B (for all PSEs for every participant, see Table S2 in Supplementary Information). These PSEs indicate that the effect of the haptic information is strong when the force pulled subjects' hand away from a contact point, and the estimated mean PSE reached nearly 4 mm for the pull-away condition. Among these PSEs, a repeated measures of ANOVA with one-within subject factors (direction of hand movement) revealed significant effect of the direction of hand movement (F(2,12) = 12.889, p = 0.01), and post hoc t-tests adjusted using Bonferroni correction to compare the effect of the direction revealed a significant difference between the data for the pull-away condition and push-back condition (p = 0.042), and between pull-away and no-move condition (p = 0.004). There was no significant difference between push-back and no-move conditions (p>0.5).

I also tested whether these PSEs are significantly different from zero by conducting one-sample t-test. This revealed that the PSE in pull-away condition was significantly different from zero (t(6) = 3.86, p = 0.008), but PSEs in neither push-back nor the no-move conditions differed significantly from zero (t(6) = 0.32, p>0.5, t(6) = 0.18, p>0.5, for push-back and no-move conditions, respectively).

These analyses indicate that hand movement affected perceived visual 3-D movement. The visually perceived direction was biased toward the direction indicated by haptic information. What is interesting in these results is the asymmetric effect of haptic information between two directions. The probability of a report of perceived protrusion decreased when the haptic device pulled the subjects' hands away from subjects, whereas the probability of a report of perceived protrusion did not increase when the device pushed their hands back. There were no difference between no-move condition and push-back condition. That is, haptic information was effective when the movement-direction was directed away from observers, but it was not effective in the reverse direction. Although this result was unexpected, several previous researchers have suggested a direction-dependent weighting in the integration of visual and proprioceptive information [10], [17]–[19]. This direction-dependent asymmetry is discussed in the General Discussion.

Experiment 1 showed that haptic information affects perception of 3-D movement. Although the direction-dependent asymmetry was unexpected, the effect was generally the same as that predicted from previous research. In the next experiment, I examined the effect of proximity of the haptic and visual information on cross-model integration. Helbig and Ernst [20] showed that prior knowledge of object identity may enable integration, even when information from vision and touch is provided at spatially discrepant locations. However, it is possible that more precise spatial coincidence is necessary to fuse the dynamic information from haptic and visual modalities. To examine whether this spatial coincidence is necessary for the integration of dynamic haptic and visual information, the cursor position was spatially separately from the hand position in Experiment 2.

## Experiment 2

The apparatus, stimuli and procedure were the same as Experiment 1 with the exception that the cursor was displayed 10 cm left to the point of the stylus. This introduces a spatial discrepancy of a 10 cm spatial separation between the visual cursor position and the haptic stylus position. Participants were the same as in Experiment 1; they participated in Experiment 2 after taking part in Experiment 1.

### Results and Discussion

**Figure 5:**
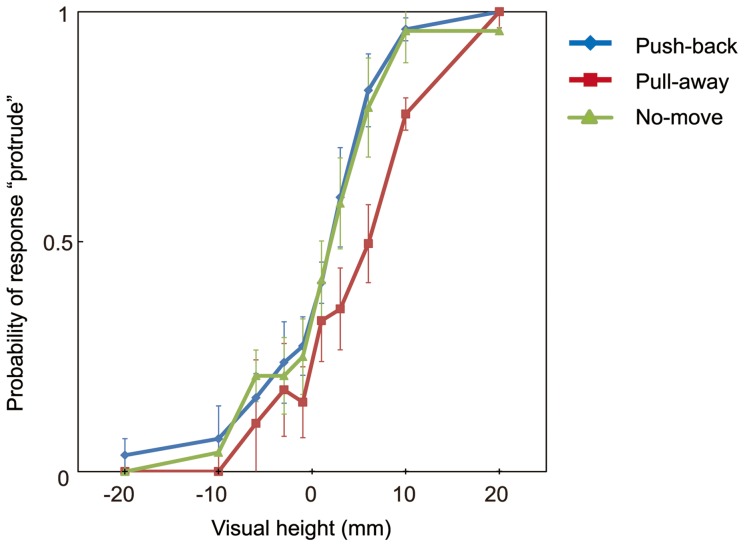
Results of Experiment 2. Results of Experiment 2 in which the cursor position was separated from the stylus position. Mean response probability over participants in each condition as a function of height.

**Figure 6:**
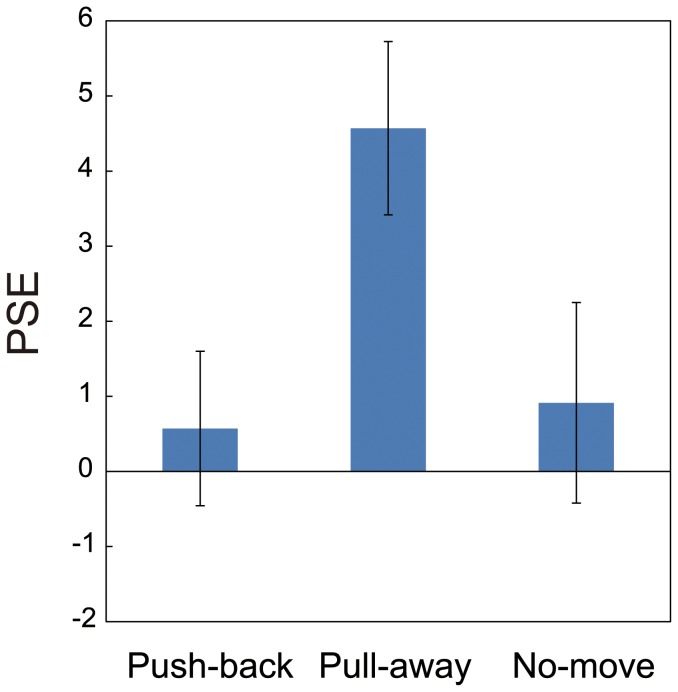
Mean of PSEs in Experiment 2. Mean of PSEs obtained from results of Experiment 2. Error bars indicate mean standard errors among participants in each of the three conditions.

Averaged probabilities of stimuli judged 'protrude' over all participants are shown in Figure 5 (for all probabilities for every participant, see Table S1). As in Experiment 1, responses from trials in which participants took less than 150 ms or longer than 400 ms to move the stylus 24 mm were omitted. The average number of omitted trails for participants in the push-back condition was 1.0 (1.3% of all the trials, SD = 2.4); this value was 1.3 (1.6% of all of the trials, SD = 2.8) for the pull-away condition. I obtained PSE by fitting cumulative Gaussian function as in Experiment 1. These values are plotted in Figure 6 (for all PSEs for every participant, see Table S2). A repeated measures of ANOVA with one-within subject factors (direction of hand movement) revealed a significant effect of the direction of hand movement (F(2,12) = 8.667, p = 0.005), and post hoc t-tests adjusted using Bonferroni correction to compare the effect of the direction revealed a significant difference between the data for the pull-away condition and push-back condition (p = 0.034). The difference between pull-away and no-move condition did not reach significance in Experiment 2 (p = 0.096). There was no significant difference between push-back and no-move conditions (p>0.5). One-sample t-tests for PSEs in each of conditions revealed that the PSE in pull-away condition was significantly different from zero (t(6) = 3.77, p = 0.009); by contrast, the PSEs in push-back and no-move conditions were not significantly different from zero (t(6) = 0.56, t(6) = 0.6, p>0.5 in both conditions).

The outcome of Experiment 2 was similar to that in Experiment 1, although the difference between pull-away and no-move condition did not attain significance in Experiment 2. These results seem to agree with the results by Helbig and Ernst [20]; however, they do not correspond to findings reported by Gepshtein et al. [21], who showed that the spatial separation between visual and haptic signals affects the combination of this information. In the results of Gepshtein et al, a 9 cm separation apparently affected the integration of information from haptic and visual modality; however, their participants were not given information about the unity of two signals. Helbig and Ernst [20] asserted that knowledge of what participants see and touch as the same object should contribute to the integration of two sensory signals. In Experiment 2 of the present study, participants knew the unity of the cursor and hand position (all participants performed in Experiment 2 after Experiment 1), and as a result, subjects could integrate two sensory signals much as in Experiment 1.

However, another question arises concerning whether or not the information from two senses are integrated even if participants recognize that two signals come from different sources. In other words, it might be the case that synchronized dynamic visual and haptic information are integrated if the sources of each of information are located within a certain spatial range. In Experiment 3, I examined whether visual perception would be affected by haptic information even if participants touched an object different from visual stimulus. If haptic information influences judgments based on visual perception when two signals are located within a certain range, the perceived height should be influenced by haptic information also in this condition. Conversely, if the cognitive identity of sources of visual and haptic information is important, the effect of haptic information would disappear.

## Experiment 3

In Experiment 3, a square (5 cm, red) was placed to the right of the center square. Participants touched the center of this lateral square instead of the concentric square at the center of the display. There was no gap between the cursor position and the point of the stylus. Because the center of the concentric square and the lateral square was 10 cm, the position of a participant's hand when s/he touched the center of the lateral square was identical to the position that participants touched the center of the concentric square in Experiment 2.

### Results and Discussion

**Figure 7:**
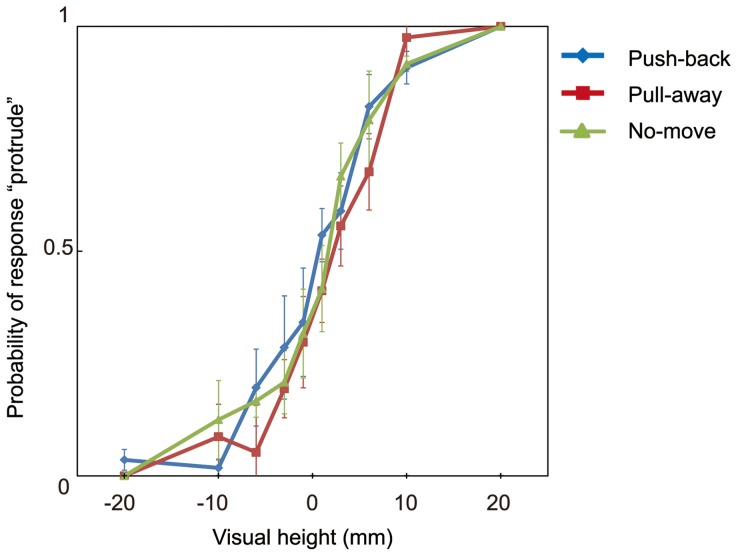
Results of Experiment 3. Results of Experiment 3 in which participants touched a different object. Means of response probabilities as function of height.

**Figure 8:**
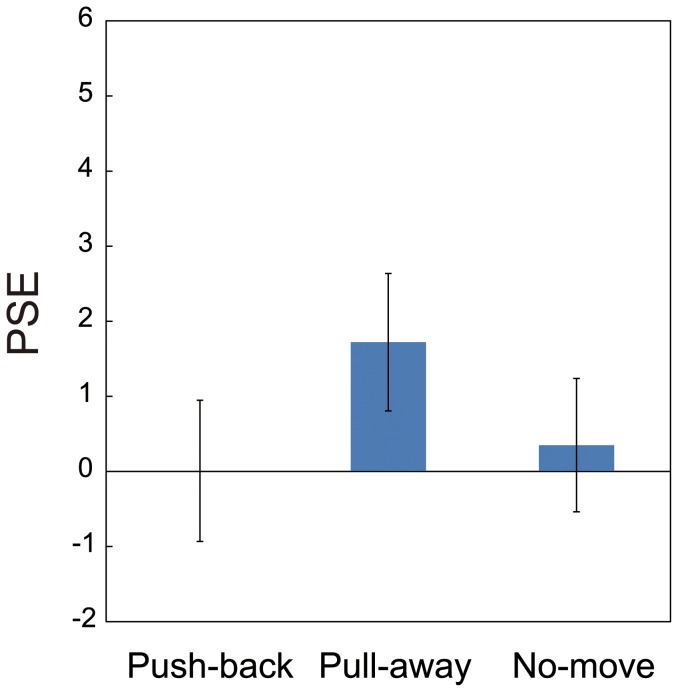
Mean of PSEs in Experiment 3. Mean of PSEs obtained from results of Experiment 3. Error bars indicate mean standard errors among participants in each of the three conditions.

Results of Experiment 3 appear in Figure 7 (for all probabilities for every participant, see Table S1). The analysis for Experiment 3 was the same as analyses of previous experiments. Responses from trials in which participants took less than 150 ms or longer than 400 ms to move the stylus 24 mm were omitted. The average number of omitted trials for participants in the push-back condition was 0.9 (1.1% of all the trials, SD = 1.5); and this value was 3.6 (4.5% of all of the trials, SD = 4.9) for the pull-away condition. PSEs obtained by fitting cumulative Gaussian function are plotted in Figure 8 (for all PSEs for every participant, see Table S2). A repeated measures ANOVA with one-within subject factors (direction of hand movement) on the PSEs revealed no significant effects of the direction of hand movement (F(2,12) = 2.736, p = .105). One-sample t-tests for PSEs in each of conditions revealed that the PSEs were not significantly different from zero in all of three conditions (push-back, t(6) = 0, p>0.5; pull-away t(6) = 1.875, p = 0.11, no-move t(6) = 1.07, p = 0.325).

If subjects made responses based only on temporally concurrent haptic information even if this arises from different events, then the probability of protrude responses should be affected by haptic information as in previous experiments. The results of the ANOVA, which revealed a non-significant main effect of direction of hand movement, and those of one-sample t-tests, which revealed that PSEs in all conditions were not significantly different from zero, indicates that the hand movement did not have an effect on visual depth perception in Experiment 3. These results are in line with control experiment in [20], in which integration of information from two senses broke down when participants were aware that two signals came from two distinct objects.

#### Analysis of merged data

To further compare the results of three experiments, I analyzed PSEs from all three experiments using a repeated measures ANOVA with two-within subject factors (direction of hand movement and experiment type). This revealed significant effect of direction of hand movement (F(2,12) = 12.039, p = 0.01) and significant interaction (F(4,24) = 3.321, p = 0.027), but no significant effect was observed for experiment type (F(2,12) = 1.446, p = .274). Post-hoc t-test with Bonferroni correction to compare the direction of hand movement revealed significant difference between the pull-away condition and no-move condition (p = 0.05), and between the pull-away and push-back conditions (p = .006). However, a significant difference was not found between the pull-away and no-move conditions (p>0.5). Because the interaction was significant, and I was interested in the difference among three experiments with the same hand-movement direction, I performed post-hoc t-test (adjusted by the Bonferroni correction) to compare effects of experiment-type in each direction of hand-movement. In the pull-away condition, the effect of the hand movement did not differ significantly between Experiment 1 and 2 (p>0.5). However, a significant difference due to experiment (Experiment 1 versus 3) was found (p = 0.033). Although a corresponding difference between Experiment 2 and 3 did not attain significance (p = 0.096), the trend was the same direction as the difference between Experiment 1 and 3. In push-back and no-move condition, there was no pair which showed significant difference (p>0.5 in all pairs).

The results of this ANOVA generally confirmed the present findings; the effect of hand-movement can be observed in the pull-away condition, and the effect of hand movement is promoted by the cognitive unity of the source of information of visual and haptic information. The difference was significant between the pull-away condition and the no-move condition. This indicates that the effect of hand-movement was direction-dependent throughout the present study. Moreover, the comparison of effects in the pull-away condition shows a significant difference between Experiment 1 and 3, and trends between Experiment 2 and 3. Although it seems that there remains slight effect of hand movement in the pull-away condition of Experiment 3 (see Figure 8), it can be said that the cognitive unity of the source of signals are important to integrate these signals.

## General Discussion

The present study reveals that in a dynamic situation, the integration of haptic information has an impact on the visual 3-D perception. Perceived depth was biased toward the depth given by the haptic device. Although classic experiments on the integration of haptic and visual information have shown 'visual capture', this phenomenon was not observed in the present experiments. That is, if visual capture were a factor then we should not find that haptic information affects visual perception. Another possible outcome was that visual and haptic information would not be integrated in a dynamic situation. In this case too we would expect to find no impact of haptic information on visual judgments. But this too did not happen. Consequently, the results are generally in accord with recent findings obtained with static situation [5], [8]–[14]. That is, the results of the present experiments can be interpreted within a framework in which weighted information from multiple modalities are integrated according to weights that reflect their respective reliabilities.

I was also interested in the impact of spatial coincidence (Experiment 2) and cognitive unity (Experiment 3) on the integration of dynamic haptic and visual information. The results showed that effects of these factors are generally as same as those reported in a previous study by Helbig and Ernst [20]. Experiment 2 showed that haptic information is effective even when the position of the cursor and hand position were spatially separated. It was assumed that more precise spatial coincidence (i.e., greater proximity) might be necessary to fuse the dynamic information from haptic and visual modalities. However, the present findings do not support this assumption. The effect of the hand movement in Experiment 2 approximated the level observed in Experiment 1. Further, the results of Experiment 3, in which the effect of hand movement was reduced in spite of the same positional relationship between hand and visual stimulus as Experiment 2, indicate that the cognitive unity of the source of visual and haptic information is important for the integration of two signals.

One interesting finding in these results involves the differential effect of hand-movement direction. Hand movement away from observer had a greater impact on perceived depth than movement toward the observer. This direction-dependent asymmetry was observed throughout three experiments. The direction-dependent weighting on the integration of visual and haptic (proprioceptive) information has been reported by several researchers [10], [17]–[19]. Assuming that direction-dependent asymmetry reflects reliability of haptic information, this finding suggests that the two movement directions differ in haptic information reliability. One explanation of such results holds that a reliance on kinesthetic information varies according to the direction of movement. The push-back condition and the pull-away condition required different proprioceptive and postural situations. van Beers et al [17] reported that the precision of proprioceptive localization of the hand differs as a function of arm movement directions Furthermore, visual and proprioceptive information are differentially weighted to efficiently use information from different modalities that have different direction-dependent precision [18] However, these studies suggest that the reliability of kinesthetic information is lower when the hand position is far from one's shoulder; this outcome is opposite to the present results. As a result, it is difficult to directly link the preceding results to the present ones. On this point, it is speculated that the reliability of haptic information from dynamic movement may differ from that derived from static positioning.

It is also possible that haptic information is more important for perceiving a concave surface than for perceiving a convex surface. One reason for this speculation is that a configuration in which surface is indented is likely to be more common than the reverse configuration. That is frequency with which humans encounter contexts in which a hand moves away from an observer concurrently with a surface indentation tends to be greater than the frequency of such contexts but the hand moves toward an observer. Speculatively, the brain might assign greater reliability to haptic information obtained from a hand moving away from an observer (i.e., than the reverse). Another possible reason is that the visual system obeys additional information from a different modality more easily when this information specifies a concave surface. It is known that the visual system is biased to perceive a convex surface especially when the visual information is ambiguous or visual object is familiar [22]–[25]. This preference for the convex could reflect prior knowledge about the distribution of surface geometry, but it would nonetheless result in a misperception in certain cases. To compensate for such a bias, the visual system might be more susceptible to additional information indicating a concave surface. Therefore, the effect of hand movement should be more pronounced in the pull-away condition. This type of integration, however, has not been previously investigated. It appears, then, that further investigation on this topic is warranted.

To summarize, three experiments demonstrate that dynamic haptic information affects 3-D visual perception. The aim of the present series of experiments was to examine the existence of the effects of haptic-visual interaction based on visual perception. Although results point to such an interaction, several questions remain. One concerns whether or not dynamic visual 3-D information has an effect on haptic perception. Another issue worthy of investigation concerns how information from two modalities are integrated when an observer 'actively' moves his/her hand. Previous research has reported that active exploration for size perception increases haptic influence [7]. It would be interesting to investigate on the role of active (intentional) movement on perception of deforming surface.

## Supporting Information

Table S1 **Probablity of response 'protrude' for every participant.**
(XLSX)

Table S2 **Obtained points of subjective equality (PSE) for every participant.**
(XLSX)
